# Diethyl 2-{[3-(2,4,6-trimethyl­benz­yl)-1-phenyl­sulfonyl-1*H*-indol-2-yl]methyl­idene}propane­dioate

**DOI:** 10.1107/S1600536810019690

**Published:** 2010-05-29

**Authors:** B. Saravanan, V. Dhayalan, A. K. Mohanakrishnan, G. Chakkaravarthi, V. Manivannan

**Affiliations:** a Department of Physics, J. J. College of Arts and Science, Pudukkottai 622 422, Tamil Nadu, India; b Department of Organic Chemistry, University of Madras, Guindy Campus, Chennai 600 025, India; c Department of Physics, CPCL Polytechnic College, Chennai 600 068, India; d Department of Research and Development, PRIST University, Vallam, Thanjavur 613 403, Tamil Nadu, India

## Abstract

In the title compound, C_32_H_33_NO_6_S, the indole ring system makes dihedral angles of 62.78 (10) and 80.53 (8)°, respectively, with the phenyl and benzene rings. In the crystal, the mol­ecules are linked through inter­molecular C—H⋯O hydrogen bonds, forming a chain along the *a* axis. Between the chains, a weak aromatic π–π stacking inter­action [centroid–centroid distance = 3.831 (2) Å] is observed.

## Related literature

For the biological activity of indole derivatives, see: Ma *et al.* (2001 ▶); Zhao *et al.* (2002 ▶); Zhou *et al.* (2006 ▶). For related structures, see: Chakkaravarthi *et al.* (2007 ▶, 2008 ▶).
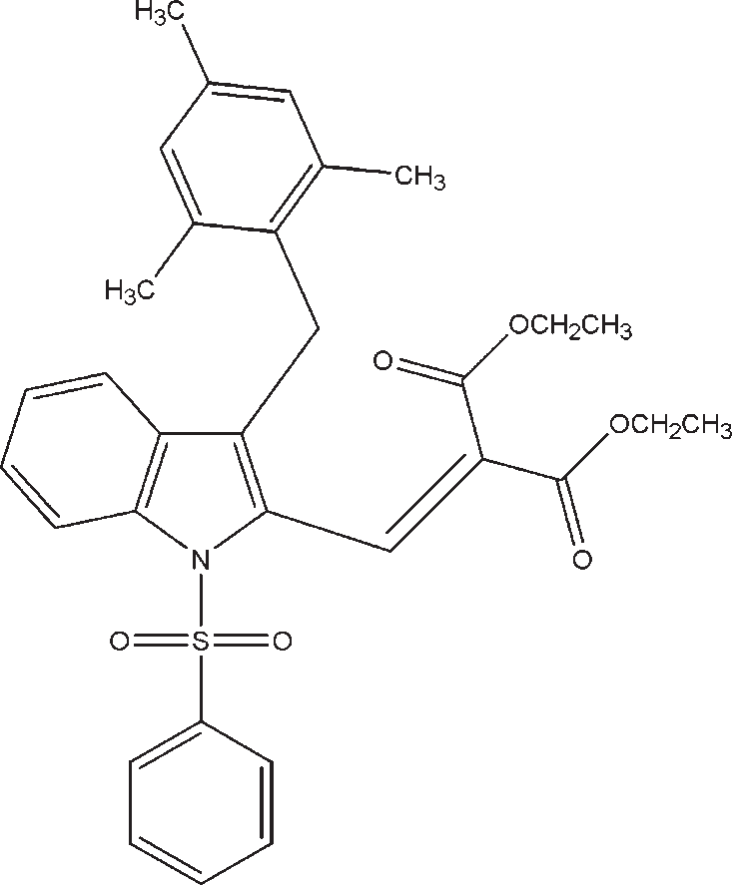


## Experimental

### 

#### Crystal data

C_32_H_33_NO_6_S
*M* *_r_* = 559.65
Triclinic, 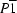

*a* = 8.5103 (4) Å
*b* = 8.9540 (4) Å
*c* = 19.6546 (10) Å
α = 78.456 (3)°
β = 87.236 (4)°
γ = 86.736 (3)°
*V* = 1463.99 (12) Å^3^
*Z* = 2
Mo *K*α radiation
μ = 0.16 mm^−1^
*T* = 295 K
0.22 × 0.18 × 0.16 mm

#### Data collection

Bruker Kappa APEXII diffractometer
Absorption correction: multi-scan (*SADABS*; Sheldrick, 1996 ▶) *T*_min_ = 0.967, *T*_max_ = 0.976
26660 measured reflections
7349 independent reflections
4328 reflections with *I* > 2σ(*I*)
*R*_int_ = 0.042

#### Refinement

*R*[*F* ^2^ > 2σ(*F* ^2^)] = 0.062
*wR*(*F* ^2^) = 0.211
*S* = 1.03
7349 reflections
370 parameters
2 restraints
H atoms treated by a mixture of independent and constrained refinement
Δρ_max_ = 0.42 e Å^−3^
Δρ_min_ = −0.31 e Å^−3^

### 

Data collection: *APEX2* (Bruker, 2004 ▶); cell refinement: *SAINT* (Bruker, 2004 ▶); data reduction: *SAINT*; program(s) used to solve structure: *SHELXS97* (Sheldrick, 2008 ▶); program(s) used to refine structure: *SHELXL97* (Sheldrick, 2008 ▶); molecular graphics: *PLATON* (Spek, 2009 ▶); software used to prepare material for publication: *SHELXL97*.

## Supplementary Material

Crystal structure: contains datablocks global, I. DOI: 10.1107/S1600536810019690/is2550sup1.cif

Structure factors: contains datablocks I. DOI: 10.1107/S1600536810019690/is2550Isup2.hkl

Additional supplementary materials:  crystallographic information; 3D view; checkCIF report

## Figures and Tables

**Table 1:** Hydrogen-bond geometry (Å, °)

*D*—H⋯*A*	*D*—H	H⋯*A*	*D*⋯*A*	*D*—H⋯*A*
C10—H10⋯O4^i^	0.93	2.43	3.179 (4)	138

Symmetry code: (i) 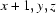.

**Fig. 1.**
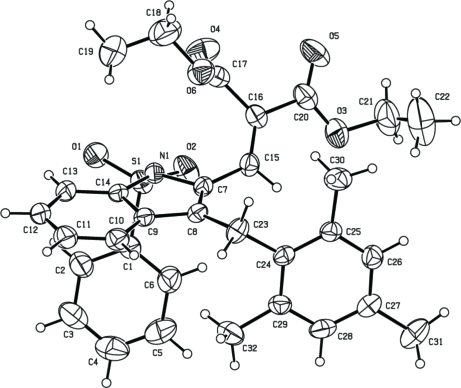
The molecular structure of (I), with atom labels and 30% probability displacement ellipsoids for non-H atoms.

**Fig. 2.**
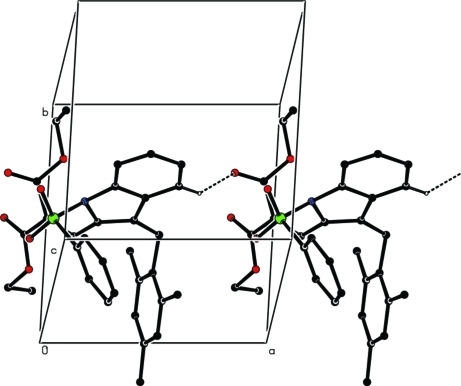
The packing of (I), viewed down the c axis. Hydrogen bonds are shown as dashed lines. H atoms not involved in hydrogen bonding have been omitted.

## supplementary crystallographic information

### Comment

Indole derivatives are found to possess anticancer, antimalarial and antihypertensive activities (Ma *et al.*, 2001; Zhou *et al.*, 2006; Zhao *et al.*, 2002). In continuation of our studies in indole derivatives, we report the crystal structure of the title compound, (I). The geometric parameters of (I) (Fig. 1) agree with those in the reported structures (Chakkaravarthi *et al.*, 2007, 2008).

The nine-membered indole ring system forms dihedral angles of 62.78 (10) and 80.53 (8)° with the phenyl ring (C1–C6) and benzene ring (C24–C29), respectively. The torsion angles O2—S1—N1—C7 and O1—S1—N1—C14 [-37.8 (2)° and 62.87 (18)°, respectively] indicate the *syn*-conformation of the sulfonyl moiety. The sum of the bond angles around N1 [342.1 (2)°] indicates that N1 is *sp*^3^-hybridized.

The molecular structure is stabilized by a weak intramolecular C—H···O interaction and the crystal packing of (I) (Fig. 2) exhibits weak intermolecular C—H···O (Table 1) and π–π interactions [*Cg*···*Cg* (-x, -y, 1-z) distance of 3.831 (2) Å]; *Cg* is the centroid of the C1–C6 ring.

### Experimental

To a solution of diethyl-2-((3-(bromomethyl)-1-(phenylsulfonyl) -1*H*-indol-2-yl)methylene)malonate (0.3 g, 0.57 mmol) in dry 1,2-dichloroethane (15 ml), anhydrous ZnBr_2_ (0.25 g, 1.11 mmol) and mesitylene (0.19 ml, 1.41 mmol) were added. It was then refluxed for 4 h under N_2_ atmosphere. The solvent was removed and the residue was quenched with ice-water (50 ml) containing 1 ml of conc. HCl, extracted with chloroform (2 × 10 ml) and dried (Na_2_SO_4_). Removal of solvent followed by flash column chromatographic purification (n-hexane/ethyl acetate 98:2) led to the isolation of product as a colourless crystal.

### Refinement

H atom attached to C15 was located from a difference Fourier map and refined freely. All other H atoms were positioned geometrically and refined using riding model, with C—H = 0.93 Å and *U*_iso_(H) = 1.2*U*_eq_(C) for aromatic C—H, C—H = 0.97 Å and *U*_iso_(H) = 1.2*U*_eq_(C) for methylene, and C—H = 0.96 Å and *U*_iso_(H) = 1.5*U*_eq_(C) for methyl. C21—C22 and C18—C19 distances were restrained to 1.550 (7) Å.

### Crystal data

**Table:**

C_32_H_33_NO_6_S	*Z* = 2
*M**_r_* = 559.65	*F*(000) = 592
Triclinic, *P*1	*D*_x_ = 1.270 Mg m^−^^3^
Hall symbol: -P 1	Mo *K*α radiation, λ = 0.71073 Å
*a* = 8.5103 (4) Å	Cell parameters from 7772 reflections
*b* = 8.9540 (4) Å	θ = 2.4–24.3°
*c* = 19.6546 (10) Å	µ = 0.16 mm^−^^1^
α = 78.456 (3)°	*T* = 295 K
β = 87.236 (4)°	Block, colourless
γ = 86.736 (3)°	0.22 × 0.18 × 0.16 mm
*V* = 1463.99 (12) Å^3^	

### Data collection

**Table:**

Bruker Kappa APEXII diffractometer	7349 independent reflections
Radiation source: fine-focus sealed tube	4328 reflections with *I* > 2σ(*I*)
graphite	*R*_int_ = 0.042
ω and φ scans	θ_max_ = 28.5°, θ_min_ = 1.1°
Absorption correction: multi-scan (*SADABS*; Sheldrick, 1996)	*h* = −11→10
*T*_min_ = 0.967, *T*_max_ = 0.976	*k* = −11→11
26660 measured reflections	*l* = −26→22

### Refinement

**Table:**

Refinement on *F*^2^	Primary atom site location: structure-invariant direct methods
Least-squares matrix: full	Secondary atom site location: difference Fourier map
*R*[*F*^2^ > 2σ(*F*^2^)] = 0.062	Hydrogen site location: inferred from neighbouring sites
*wR*(*F*^2^) = 0.211	H atoms treated by a mixture of independent and constrained refinement
*S* = 1.03	*w* = 1/[σ^2^(*F*_o_^2^) + (0.105*P*)^2^ + 0.4069*P*] where *P* = (*F*_o_^2^ + 2*F*_c_^2^)/3
7349 reflections	(Δ/σ)_max_ < 0.001
370 parameters	Δρ_max_ = 0.42 e Å^−^^3^
2 restraints	Δρ_min_ = −0.31 e Å^−^^3^

### Fractional atomic coordinates and isotropic or equivalent isotropic displacement parameters (Å^2^)

**Table:**

	*x*	*y*	*z*	*U*_iso_*/*U*_eq_	
S1	−0.00108 (7)	0.35985 (9)	0.37321 (3)	0.0626 (2)	
O1	−0.0678 (2)	0.4651 (3)	0.41334 (11)	0.0837 (6)	
O2	−0.0971 (2)	0.2939 (3)	0.33112 (10)	0.0790 (6)	
O3	−0.0750 (3)	0.2569 (3)	0.11855 (14)	0.1099 (9)	
O4	−0.1997 (3)	0.6190 (3)	0.21742 (15)	0.1050 (8)	
O5	−0.1945 (3)	0.4863 (3)	0.08992 (14)	0.1139 (9)	
O6	0.0438 (3)	0.6904 (2)	0.18672 (12)	0.0852 (6)	
N1	0.1367 (2)	0.4543 (2)	0.31990 (10)	0.0526 (5)	
C1	0.1061 (3)	0.2144 (3)	0.42788 (13)	0.0598 (6)	
C2	0.1303 (4)	0.2243 (4)	0.49532 (16)	0.0859 (9)	
H2	0.0909	0.3089	0.5126	0.103*	
C3	0.2130 (5)	0.1083 (5)	0.5370 (2)	0.1025 (12)	
H3	0.2274	0.1134	0.5831	0.123*	
C4	0.2739 (5)	−0.0135 (5)	0.5118 (2)	0.1069 (13)	
H4	0.3293	−0.0919	0.5406	0.128*	
C5	0.2542 (6)	−0.0217 (4)	0.4438 (3)	0.1226 (16)	
H5	0.2988	−0.1040	0.4261	0.147*	
C6	0.1677 (5)	0.0930 (3)	0.40152 (18)	0.0889 (10)	
H6	0.1518	0.0873	0.3556	0.107*	
C7	0.2050 (3)	0.3920 (3)	0.26267 (12)	0.0489 (5)	
C8	0.3634 (3)	0.4001 (2)	0.26078 (12)	0.0487 (5)	
C9	0.4031 (3)	0.4704 (2)	0.31715 (12)	0.0490 (5)	
C10	0.5466 (3)	0.5094 (3)	0.33863 (15)	0.0623 (6)	
H10	0.6403	0.4902	0.3148	0.075*	
C11	0.5468 (4)	0.5764 (3)	0.39536 (16)	0.0722 (8)	
H11	0.6416	0.6032	0.4102	0.087*	
C12	0.4072 (4)	0.6049 (3)	0.43112 (16)	0.0721 (8)	
H12	0.4106	0.6489	0.4700	0.087*	
C13	0.2652 (3)	0.5699 (3)	0.41068 (14)	0.0653 (7)	
H13	0.1721	0.5906	0.4346	0.078*	
C14	0.2644 (3)	0.5025 (3)	0.35296 (12)	0.0503 (5)	
C15	0.1071 (3)	0.3562 (3)	0.20960 (13)	0.0563 (6)	
C16	−0.0135 (3)	0.4425 (3)	0.18005 (13)	0.0592 (6)	
C17	−0.0687 (4)	0.5916 (3)	0.19722 (14)	0.0671 (7)	
C18	0.0018 (6)	0.8430 (4)	0.1979 (2)	0.1203 (15)	
H19A	−0.1097	0.8655	0.1904	0.144*	
H19B	0.0600	0.9163	0.1644	0.144*	
C19	0.0364 (8)	0.8585 (6)	0.2684 (3)	0.161 (2)	
H66A	−0.0292	0.7935	0.3014	0.241*	
H66B	0.0159	0.9626	0.2730	0.241*	
H66C	0.1451	0.8294	0.2769	0.241*	
C20	−0.1044 (4)	0.4002 (4)	0.12446 (16)	0.0763 (8)	
C21	−0.1577 (6)	0.2046 (7)	0.0635 (2)	0.1310 (17)	
H21A	−0.1874	0.0999	0.0792	0.157*	
H21B	−0.2520	0.2685	0.0512	0.157*	
C22	−0.0463 (8)	0.2163 (10)	0.0041 (3)	0.191 (3)	
H33A	0.0557	0.1769	0.0198	0.287*	
H33B	−0.0402	0.3214	−0.0185	0.287*	
H33C	−0.0812	0.1584	−0.0280	0.287*	
C23	0.4831 (3)	0.3629 (3)	0.20674 (15)	0.0629 (7)	
H23A	0.5870	0.3602	0.2253	0.076*	
H23B	0.4785	0.4456	0.1665	0.076*	
C24	0.4654 (3)	0.2139 (3)	0.18243 (12)	0.0514 (5)	
C25	0.4139 (3)	0.2122 (3)	0.11623 (12)	0.0545 (6)	
C26	0.4067 (3)	0.0728 (3)	0.09579 (14)	0.0637 (7)	
H26	0.3716	0.0719	0.0518	0.076*	
C27	0.4493 (3)	−0.0634 (3)	0.13816 (16)	0.0683 (7)	
C28	0.5004 (4)	−0.0589 (3)	0.20301 (16)	0.0722 (8)	
H28	0.5302	−0.1500	0.2324	0.087*	
C29	0.5091 (3)	0.0768 (3)	0.22612 (13)	0.0606 (6)	
C30	0.3701 (4)	0.3567 (3)	0.06561 (15)	0.0847 (9)	
H30A	0.4612	0.4166	0.0538	0.127*	
H30B	0.2894	0.4142	0.0865	0.127*	
H30C	0.3319	0.3315	0.0244	0.127*	
C31	0.4440 (5)	−0.2144 (4)	0.1150 (2)	0.1061 (13)	
H31A	0.5475	−0.2632	0.1164	0.159*	
H31B	0.4078	−0.1970	0.0685	0.159*	
H31C	0.3732	−0.2789	0.1456	0.159*	
C32	0.5710 (5)	0.0727 (5)	0.29715 (17)	0.0964 (11)	
H32A	0.5875	−0.0314	0.3207	0.145*	
H32B	0.4960	0.1244	0.3236	0.145*	
H32C	0.6690	0.1227	0.2924	0.145*	
H15	0.143 (4)	0.268 (4)	0.1932 (16)	0.085 (9)*	

### Atomic displacement parameters (Å^2^)

**Table:**

	*U*^11^	*U*^22^	*U*^33^	*U*^12^	*U*^13^	*U*^23^
S1	0.0375 (3)	0.0952 (5)	0.0569 (4)	0.0061 (3)	−0.0024 (3)	−0.0216 (3)
O1	0.0545 (11)	0.1244 (17)	0.0742 (13)	0.0296 (11)	0.0046 (9)	−0.0356 (12)
O2	0.0433 (10)	0.1292 (17)	0.0664 (11)	−0.0156 (10)	−0.0065 (8)	−0.0196 (12)
O3	0.116 (2)	0.123 (2)	0.1081 (18)	0.0088 (16)	−0.0507 (16)	−0.0572 (16)
O4	0.0776 (16)	0.0955 (16)	0.132 (2)	0.0165 (13)	0.0206 (15)	−0.0106 (15)
O5	0.114 (2)	0.134 (2)	0.0925 (17)	0.0064 (17)	−0.0521 (16)	−0.0129 (16)
O6	0.0803 (14)	0.0767 (13)	0.1012 (16)	−0.0048 (11)	−0.0096 (12)	−0.0222 (12)
N1	0.0419 (10)	0.0665 (12)	0.0531 (11)	0.0079 (9)	−0.0069 (8)	−0.0224 (9)
C1	0.0464 (13)	0.0751 (16)	0.0592 (14)	−0.0097 (12)	−0.0018 (11)	−0.0144 (12)
C2	0.082 (2)	0.112 (2)	0.0647 (18)	0.0148 (19)	−0.0113 (16)	−0.0244 (18)
C3	0.100 (3)	0.126 (3)	0.076 (2)	0.005 (2)	−0.025 (2)	−0.003 (2)
C4	0.112 (3)	0.079 (2)	0.117 (3)	−0.005 (2)	−0.026 (3)	0.016 (2)
C5	0.171 (5)	0.0572 (19)	0.136 (4)	0.013 (2)	−0.016 (3)	−0.014 (2)
C6	0.126 (3)	0.0638 (17)	0.080 (2)	−0.0078 (18)	−0.013 (2)	−0.0185 (16)
C7	0.0446 (12)	0.0520 (12)	0.0521 (12)	0.0033 (9)	−0.0016 (10)	−0.0168 (10)
C8	0.0460 (12)	0.0451 (11)	0.0557 (13)	0.0033 (9)	−0.0004 (10)	−0.0132 (10)
C9	0.0453 (12)	0.0443 (11)	0.0578 (13)	0.0030 (9)	−0.0075 (10)	−0.0111 (10)
C10	0.0492 (14)	0.0630 (15)	0.0762 (17)	−0.0023 (11)	−0.0088 (12)	−0.0160 (13)
C11	0.0664 (18)	0.0683 (16)	0.087 (2)	−0.0012 (13)	−0.0280 (15)	−0.0228 (15)
C12	0.081 (2)	0.0662 (16)	0.0774 (18)	0.0166 (14)	−0.0329 (16)	−0.0323 (14)
C13	0.0630 (16)	0.0722 (16)	0.0663 (16)	0.0201 (13)	−0.0149 (13)	−0.0305 (13)
C14	0.0471 (12)	0.0492 (12)	0.0571 (13)	0.0112 (10)	−0.0116 (10)	−0.0175 (10)
C15	0.0494 (13)	0.0685 (15)	0.0556 (14)	−0.0023 (11)	0.0002 (11)	−0.0243 (12)
C16	0.0516 (14)	0.0766 (16)	0.0496 (13)	−0.0056 (12)	−0.0022 (11)	−0.0121 (12)
C17	0.0627 (17)	0.0747 (17)	0.0579 (15)	0.0066 (14)	−0.0037 (13)	−0.0016 (13)
C18	0.129 (4)	0.080 (2)	0.152 (4)	0.010 (2)	−0.020 (3)	−0.025 (3)
C19	0.221 (7)	0.123 (4)	0.158 (5)	0.045 (4)	−0.067 (5)	−0.075 (3)
C20	0.0659 (18)	0.100 (2)	0.0638 (17)	−0.0061 (16)	−0.0127 (14)	−0.0151 (16)
C21	0.119 (4)	0.178 (5)	0.116 (3)	−0.015 (3)	−0.037 (3)	−0.069 (3)
C22	0.170 (6)	0.307 (9)	0.127 (4)	−0.039 (6)	−0.001 (4)	−0.109 (6)
C23	0.0544 (14)	0.0630 (14)	0.0751 (17)	−0.0084 (12)	0.0160 (12)	−0.0250 (13)
C24	0.0454 (12)	0.0514 (12)	0.0561 (13)	0.0037 (10)	0.0065 (10)	−0.0116 (10)
C25	0.0534 (13)	0.0565 (13)	0.0496 (13)	0.0071 (10)	0.0074 (10)	−0.0058 (10)
C26	0.0599 (15)	0.0753 (17)	0.0586 (15)	0.0010 (13)	0.0005 (12)	−0.0217 (13)
C27	0.0668 (17)	0.0553 (14)	0.0840 (19)	0.0031 (12)	0.0043 (14)	−0.0204 (14)
C28	0.0753 (19)	0.0520 (14)	0.082 (2)	0.0174 (13)	−0.0007 (15)	−0.0013 (13)
C29	0.0551 (14)	0.0650 (15)	0.0589 (15)	0.0131 (12)	−0.0055 (11)	−0.0092 (12)
C30	0.106 (3)	0.0739 (18)	0.0615 (17)	0.0176 (17)	0.0107 (16)	0.0080 (14)
C31	0.113 (3)	0.0680 (19)	0.146 (3)	−0.0031 (19)	0.008 (3)	−0.046 (2)
C32	0.096 (3)	0.117 (3)	0.074 (2)	0.029 (2)	−0.0270 (18)	−0.0164 (19)

### Geometric parameters (Å, °)

**Table:**

S1—O2	1.420 (2)	C15—H15	0.94 (3)
S1—O1	1.424 (2)	C16—C17	1.487 (4)
S1—N1	1.682 (2)	C16—C20	1.488 (4)
S1—C1	1.755 (3)	C18—C19	1.465 (5)
O3—C20	1.320 (4)	C18—H19A	0.9700
O3—C21	1.483 (4)	C18—H19B	0.9700
O4—C17	1.194 (4)	C19—H66A	0.9600
O5—C20	1.190 (4)	C19—H66B	0.9600
O6—C17	1.321 (4)	C19—H66C	0.9600
O6—C18	1.449 (4)	C21—C22	1.458 (5)
N1—C14	1.422 (3)	C21—H21A	0.9700
N1—C7	1.438 (3)	C21—H21B	0.9700
C1—C6	1.363 (4)	C22—H33A	0.9600
C1—C2	1.372 (4)	C22—H33B	0.9600
C2—C3	1.370 (5)	C22—H33C	0.9600
C2—H2	0.9300	C23—C24	1.522 (3)
C3—C4	1.354 (6)	C23—H23A	0.9700
C3—H3	0.9300	C23—H23B	0.9700
C4—C5	1.371 (6)	C24—C29	1.392 (3)
C4—H4	0.9300	C24—C25	1.396 (3)
C5—C6	1.386 (5)	C25—C26	1.391 (3)
C5—H5	0.9300	C25—C30	1.506 (4)
C6—H6	0.9300	C26—C27	1.373 (4)
C7—C8	1.353 (3)	C26—H26	0.9300
C7—C15	1.460 (3)	C27—C28	1.375 (4)
C8—C9	1.441 (3)	C27—C31	1.514 (4)
C8—C23	1.508 (3)	C28—C29	1.387 (4)
C9—C14	1.391 (3)	C28—H28	0.9300
C9—C10	1.396 (3)	C29—C32	1.509 (4)
C10—C11	1.368 (4)	C30—H30A	0.9600
C10—H10	0.9300	C30—H30B	0.9600
C11—C12	1.389 (4)	C30—H30C	0.9600
C11—H11	0.9300	C31—H31A	0.9600
C12—C13	1.364 (4)	C31—H31B	0.9600
C12—H12	0.9300	C31—H31C	0.9600
C13—C14	1.388 (3)	C32—H32A	0.9600
C13—H13	0.9300	C32—H32B	0.9600
C15—C16	1.332 (4)	C32—H32C	0.9600
			
O2—S1—O1	120.54 (13)	C19—C18—H19B	109.4
O2—S1—N1	106.83 (11)	H19A—C18—H19B	108.0
O1—S1—N1	105.76 (13)	C18—C19—H66A	109.5
O2—S1—C1	109.32 (13)	C18—C19—H66B	109.5
O1—S1—C1	108.79 (13)	H66A—C19—H66B	109.5
N1—S1—C1	104.36 (11)	C18—C19—H66C	109.5
C20—O3—C21	116.6 (3)	H66A—C19—H66C	109.5
C17—O6—C18	117.1 (3)	H66B—C19—H66C	109.5
C14—N1—C7	106.24 (18)	O5—C20—O3	124.2 (3)
C14—N1—S1	115.80 (16)	O5—C20—C16	123.4 (3)
C7—N1—S1	120.08 (16)	O3—C20—C16	112.5 (3)
C6—C1—C2	120.7 (3)	C22—C21—O3	105.9 (4)
C6—C1—S1	118.6 (2)	C22—C21—H21A	110.5
C2—C1—S1	120.7 (2)	O3—C21—H21A	110.5
C3—C2—C1	119.4 (3)	C22—C21—H21B	110.5
C3—C2—H2	120.3	O3—C21—H21B	110.5
C1—C2—H2	120.3	H21A—C21—H21B	108.7
C4—C3—C2	120.6 (4)	C21—C22—H33A	109.5
C4—C3—H3	119.7	C21—C22—H33B	109.5
C2—C3—H3	119.7	H33A—C22—H33B	109.5
C3—C4—C5	120.2 (4)	C21—C22—H33C	109.5
C3—C4—H4	119.9	H33A—C22—H33C	109.5
C5—C4—H4	119.9	H33B—C22—H33C	109.5
C4—C5—C6	119.7 (4)	C8—C23—C24	116.5 (2)
C4—C5—H5	120.1	C8—C23—H23A	108.2
C6—C5—H5	120.1	C24—C23—H23A	108.2
C1—C6—C5	119.3 (3)	C8—C23—H23B	108.2
C1—C6—H6	120.4	C24—C23—H23B	108.2
C5—C6—H6	120.4	H23A—C23—H23B	107.3
C8—C7—N1	109.75 (19)	C29—C24—C25	119.5 (2)
C8—C7—C15	128.0 (2)	C29—C24—C23	119.0 (2)
N1—C7—C15	121.3 (2)	C25—C24—C23	121.5 (2)
C7—C8—C9	107.7 (2)	C26—C25—C24	118.9 (2)
C7—C8—C23	128.9 (2)	C26—C25—C30	119.0 (2)
C9—C8—C23	123.0 (2)	C24—C25—C30	122.1 (2)
C14—C9—C10	119.5 (2)	C27—C26—C25	122.4 (3)
C14—C9—C8	108.2 (2)	C27—C26—H26	118.8
C10—C9—C8	132.3 (2)	C25—C26—H26	118.8
C11—C10—C9	118.7 (3)	C26—C27—C28	117.6 (2)
C11—C10—H10	120.6	C26—C27—C31	122.1 (3)
C9—C10—H10	120.6	C28—C27—C31	120.2 (3)
C10—C11—C12	120.8 (3)	C27—C28—C29	122.3 (2)
C10—C11—H11	119.6	C27—C28—H28	118.9
C12—C11—H11	119.6	C29—C28—H28	118.9
C13—C12—C11	121.7 (3)	C28—C29—C24	119.3 (2)
C13—C12—H12	119.1	C28—C29—C32	119.1 (3)
C11—C12—H12	119.1	C24—C29—C32	121.6 (3)
C12—C13—C14	117.7 (3)	C25—C30—H30A	109.5
C12—C13—H13	121.1	C25—C30—H30B	109.5
C14—C13—H13	121.1	H30A—C30—H30B	109.5
C13—C14—C9	121.5 (2)	C25—C30—H30C	109.5
C13—C14—N1	130.4 (2)	H30A—C30—H30C	109.5
C9—C14—N1	108.11 (19)	H30B—C30—H30C	109.5
C16—C15—C7	126.1 (2)	C27—C31—H31A	109.5
C16—C15—H15	120.2 (19)	C27—C31—H31B	109.5
C7—C15—H15	113.4 (19)	H31A—C31—H31B	109.5
C15—C16—C17	124.2 (2)	C27—C31—H31C	109.5
C15—C16—C20	122.8 (3)	H31A—C31—H31C	109.5
C17—C16—C20	113.0 (2)	H31B—C31—H31C	109.5
O4—C17—O6	124.2 (3)	C29—C32—H32A	109.5
O4—C17—C16	123.9 (3)	C29—C32—H32B	109.5
O6—C17—C16	112.0 (2)	H32A—C32—H32B	109.5
O6—C18—C19	111.3 (4)	C29—C32—H32C	109.5
O6—C18—H19A	109.4	H32A—C32—H32C	109.5
C19—C18—H19A	109.4	H32B—C32—H32C	109.5
O6—C18—H19B	109.4		
			
O2—S1—N1—C14	−167.56 (16)	C7—N1—C14—C13	−179.8 (3)
O1—S1—N1—C14	62.87 (18)	S1—N1—C14—C13	−43.7 (3)
C1—S1—N1—C14	−51.82 (18)	C7—N1—C14—C9	0.0 (2)
O2—S1—N1—C7	−37.8 (2)	S1—N1—C14—C9	136.08 (18)
O1—S1—N1—C7	−167.42 (17)	C8—C7—C15—C16	−124.3 (3)
C1—S1—N1—C7	77.89 (19)	N1—C7—C15—C16	42.8 (4)
O2—S1—C1—C6	40.3 (3)	C7—C15—C16—C17	−0.3 (4)
O1—S1—C1—C6	173.8 (2)	C7—C15—C16—C20	177.9 (2)
N1—S1—C1—C6	−73.7 (3)	C18—O6—C17—O4	−2.7 (5)
O2—S1—C1—C2	−141.1 (2)	C18—O6—C17—C16	176.6 (3)
O1—S1—C1—C2	−7.6 (3)	C15—C16—C17—O4	−122.4 (3)
N1—S1—C1—C2	104.9 (3)	C20—C16—C17—O4	59.2 (4)
C6—C1—C2—C3	−2.1 (5)	C15—C16—C17—O6	58.3 (4)
S1—C1—C2—C3	179.4 (3)	C20—C16—C17—O6	−120.1 (3)
C1—C2—C3—C4	1.6 (6)	C17—O6—C18—C19	94.3 (5)
C2—C3—C4—C5	0.4 (7)	C21—O3—C20—O5	1.9 (6)
C3—C4—C5—C6	−1.9 (7)	C21—O3—C20—C16	−178.8 (3)
C2—C1—C6—C5	0.6 (5)	C15—C16—C20—O5	−167.4 (3)
S1—C1—C6—C5	179.2 (3)	C17—C16—C20—O5	11.1 (4)
C4—C5—C6—C1	1.4 (7)	C15—C16—C20—O3	13.4 (4)
C14—N1—C7—C8	0.4 (3)	C17—C16—C20—O3	−168.2 (3)
S1—N1—C7—C8	−133.46 (18)	C20—O3—C21—C22	97.3 (6)
C14—N1—C7—C15	−168.9 (2)	C7—C8—C23—C24	−46.2 (4)
S1—N1—C7—C15	57.3 (3)	C9—C8—C23—C24	141.6 (2)
N1—C7—C8—C9	−0.6 (3)	C8—C23—C24—C29	−74.8 (3)
C15—C7—C8—C9	167.8 (2)	C8—C23—C24—C25	108.4 (3)
N1—C7—C8—C23	−173.7 (2)	C29—C24—C25—C26	0.4 (4)
C15—C7—C8—C23	−5.4 (4)	C23—C24—C25—C26	177.2 (2)
C7—C8—C9—C14	0.5 (3)	C29—C24—C25—C30	−177.9 (2)
C23—C8—C9—C14	174.2 (2)	C23—C24—C25—C30	−1.1 (4)
C7—C8—C9—C10	−178.5 (2)	C24—C25—C26—C27	−0.5 (4)
C23—C8—C9—C10	−4.9 (4)	C30—C25—C26—C27	177.9 (3)
C14—C9—C10—C11	1.0 (4)	C25—C26—C27—C28	0.1 (4)
C8—C9—C10—C11	−180.0 (2)	C25—C26—C27—C31	−178.8 (3)
C9—C10—C11—C12	0.2 (4)	C26—C27—C28—C29	0.3 (4)
C10—C11—C12—C13	−1.1 (5)	C31—C27—C28—C29	179.3 (3)
C11—C12—C13—C14	0.8 (4)	C27—C28—C29—C24	−0.3 (4)
C12—C13—C14—C9	0.4 (4)	C27—C28—C29—C32	−178.2 (3)
C12—C13—C14—N1	−179.9 (2)	C25—C24—C29—C28	0.0 (4)
C10—C9—C14—C13	−1.3 (4)	C23—C24—C29—C28	−176.9 (2)
C8—C9—C14—C13	179.5 (2)	C25—C24—C29—C32	177.8 (3)
C10—C9—C14—N1	178.9 (2)	C23—C24—C29—C32	0.9 (4)
C8—C9—C14—N1	−0.3 (3)		

### Hydrogen-bond geometry (Å, °)

**Table:**

*D*—H···*A*	*D*—H	H···*A*	*D*···*A*	*D*—H···*A*
C10—H10···O4^i^	0.93	2.43	3.179 (4)	138
C13—H13···O1	0.93	2.48	3.030 (4)	118

Symmetry codes: (i) *x*+1, *y*, *z*.

## References

[bb1] Bruker (2004). *APEX2* Bruker AXS Inc., Madison, Wisconsin, USA.

[bb2] Chakkaravarthi, G., Dhayalan, V., Mohanakrishnan, A. K. & Manivannan, V. (2007). *Acta Cryst.* E**63**, o3698.

[bb3] Chakkaravarthi, G., Dhayalan, V., Mohanakrishnan, A. K. & Manivannan, V. (2008). *Acta Cryst.* E**64**, o542. 10.1107/S1600536808003024 PMC2960400 21201561

[bb4] Ma, C., Liu, X., Li, X., Flippen-Anderson, J., Yu, S. & Cook, J. M. (2001). *J. Org. Chem.* **66**, 4525–4542. 10.1021/jo001679s 11421771

[bb5] Sheldrick, G. M. (1996). *SADABS* University of Göttingen, Germany.

[bb6] Sheldrick, G. M. (2008). *Acta Cryst.* A**64**, 112–122. 10.1107/S0108767307043930 18156677

[bb7] Spek, A. L. (2009). *Acta Cryst.* D**65**, 148–155. 10.1107/S090744490804362X PMC2631630 19171970

[bb8] Zhao, S., Liao, X. & Cook, J. M. (2002). *Org. Lett.* **4**, 687–690. 10.1021/ol010222h 11869102

[bb9] Zhou, H., Liao, X., Yin, W., Ma, J. & Cook, J. M. (2006). *J. Org. Chem.* **71**, 251–259. 10.1021/jo052081t 16388644

